# Increased dynamics in the 40–57 Ω-loop of the G41S variant of human cytochrome *c* promote its pro-apoptotic conformation

**DOI:** 10.1038/srep30447

**Published:** 2016-07-27

**Authors:** Andreas Ioannis Karsisiotis, Oliver M. Deacon, Michael T. Wilson, Colin Macdonald, Tharin M. A. Blumenschein, Geoffrey R. Moore, Jonathan A. R. Worrall

**Affiliations:** 1 School of Biological Sciences, University of Essex, Wivenhoe Park, Colchester, CO4 3SQ, UK; 2 School of Chemistry, University of East Anglia, Norwich Research Park, NR4 7TJ, UK

## Abstract

Thrombocytopenia 4 is an inherited autosomal dominant thrombocytopenia, which occurs due to mutations in the human gene for cytochrome *c* that results in enhanced mitochondrial apoptotic activity. The Gly41Ser mutation was the first to be reported. Here we report stopped-flow kinetic studies of azide binding to human ferricytochrome *c* and its Gly41Ser variant, together with backbone amide H/D exchange and ^15^N-relaxation dynamics using NMR spectroscopy, to show that alternative conformations are kinetically and thermodynamically more readily accessible for the Gly41Ser variant than for the wild-type protein. Our work reveals a direct conformational link between the 40–57 Ω-loop in which residue 41 resides and the dynamical properties of the axial ligand to the heme iron, Met80, such that the replacement of glycine by serine promotes the dissociation of the Met80 ligand, thereby increasing the population of a peroxidase active state, which is a key non-native conformational state in apoptosis.

Mitochondrial cytochrome *c* (cyt) is a multifunctional protein that can act as an electron carrier in oxidative phosphorylation as a peroxidase in the early stages of the intrinsic apoptosis pathway, as a component of the apoptosome, a key complex in the apoptotic pathway and as a signalling molecule affecting the activity of the nuclear oncoprotein SET/template-activating factor-Iβ (SET/TAF-Iβ) complex^1^,^2^,^3^,^4^. Cyt possesses a hexacoordinate heme iron, where His18 and Met80 serve as the axial heme iron ligands, with the heme encapsulated in a hydrophobic environment created by a polypeptide fold of five α-helices and 3 Ω-loops (Fig. 1A). Under native conditions ferricyt possesses a low level of peroxidase activity^5^ due to the existence of an equilibrium between the dominant hexacoordinate native form, and a minor populated pentacoordinate form in which Met80 is not coordinated to the heme iron^6^. Preceding its release from the mitochondrion at the onset of apoptosis, cyt forms a complex with the phospholipid cardiolipin (CL), which drives the formation of the pentacoordinate form and greatly boosts peroxidase activity^2^,^7^. Further to this, at alkaline pH, ferricyt can access a conformational state whereby a Lys residue (73 or 79) within the 71–85 Ω-loop replaces the native Met80 ligand^8^. This alkaline isomerisation occurs with an apparent pK of ~8–9, depending on solution conditions and temperature^9^. Thus ferricyt has several energetically accessible conformational states with very different properties from the native state.

Thrombocytopenia 4 (THC4; OMIM 612004) is an inherited autosomal disease, which occurs due to mutations in the human cytochrome *c* gene (*CYCS*) resulting in enhanced mitochondrial apoptotic activity^10^. Two mutations in the *CYCS* gene have been identified that produce the G41S and Y48H variants of human cytochrome *c* (H-cyt)^10^,^11^. The G41S and Y48H substitutions are located in the 40–57 Ω-loop^12^,^13^,^14^,^15^, which has the lowest free energy of the five cooperative folding/unfolding units (foldons) assigned in cyt^16^,^17^ (Fig. 1A). In the absence of CL the G41S variant has been shown to have increased peroxidase activity relative to the wild-type (WT) protein^18^,^19^. High-resolution X-ray structures for the WT and G41S variant of H-cyt have been determined^20^,^21^ with no gross structural differences between them detected. However, subtle differences in a hydrogen-bond network in the vicinity of the heme propionate-7 substituent were observed^21^ (Fig. 1B) and these may be significant as the electronic properties of the heme propionates are known to affect heme reactivity^22^. The increase in polarity of the heme environment of the G41S protein resulting from the movement of Arg38 and the incorporation of additional water molecules (Fig. 1B) may also contribute to an ease of access of H_2_O_2_ to the heme of the G41S variant leading directly to its enhanced peroxidase activity^19^,^23^. In addition to the small conformational difference between WT and the G41S variant there is a substantial difference in global stability with the free energy of denaturation of WT H-ferricyt at 10.5 kcal mol^−1^ being 2.6 kcal mol^−1^ greater than that of the G41S variant^21^.

Based on the inherent ability of ferricyt to access non-native conformations, it is a possibility that the G41S variant perturbs the dynamics of the H-ferricyt energy landscape, thereby changing the relative populations and kinetics of conversion between the native state and a non-native conformer. Solution state NMR spectroscopy is well suited to provide insight at atomic resolution on the dynamics and conformational changes that serve to regulate function in biomolecules^24^,^25^. Past NMR investigations have probed the backbone dynamics of cyt with the rationale of assessing whether differences in dynamics exist between the heme redox states^26^,^27^,^28^. In addition to NMR the dynamics of cyt are open to investigation by a variety of heme-centred approaches, particularly with ligand binding studies^6^,^29^. From such studies, combined with NMR data, a consensus exists that the energy landscapes of mitochondrial cyt differ considerably between redox states with the ferric form being more flexible^22^,^27^ and having a lower stability than the ferrous form^22^,^30^. This may have functional implications for cyt function in apoptosis, as it is the ferric oxidation state that has been demonstrated to be necessary for interaction with key components of the apoptotic pathway such as the Apoptotic protease activating factor 1 (Apaf-1)^31^ and also to facilitate peroxidase activity^32^.

In the present study we have used azide (N_3_^−^) binding to the heme iron of H-ferricyt, backbone amide proton H/D exchange and ^15^N relaxation dynamics of the oxidised protein to assess whether the G41S variant has altered dynamics compared to WT H-ferricyt. We find that the population of the pentacoordinate form is significantly greater for the G41S variant than the WT protein, and that the G41S variant has distinctly enhanced dynamics, particularly in the 40–57 Ω-loop foldon, which give rise to the increased lability of the Met80 ligand. These findings offer a direct structural insight into how main chain dynamics relate to key structural properties of the heme iron of H-ferricyt and importantly how the G41S variant is proapoptotic^10^,^19^,^21^,^23^ in the context of thrombocytopenia 4.

## Results and Discussion

### The pentacoordinate heme form is more populated in the G41S variant

Exogenous ligand binding to ferricyt can be used as a diagnostic probe to assess the lability of the Met80 ligand. On mixing N_3_^−^ with WT or G41S H-ferricyt an optical transition was observed (Fig. 2A) with differing time courses for the two proteins, as shown in the inset to Fig. 2A. Such an optical transition reflects the dissociation of the Met80 ligand from the heme and the binding of N_3_^−^ in its place. Confirmation of this assignment was obtained in separate experiments at higher [H-ferricyt] (Fig. S1) that revealed the addition of N_3_^−^ to WT and G41S led to the bleaching of the 695 nm band, which is diagnostic for the loss of the Met80 ligand. The optical transition consisted of a single exponential at every wavelength with no intermediates detected (inset Fig. 2A). The normalised amplitudes of such time courses, taken over a range of [N_3_^−^] conform to a simple hyperbolic binding isotherm (Fig. 2B) to yield apparent equilibrium dissociation constants (*K*_app_) of 0.31 ± 0.03 and 0.11 ± 0.01 M for WT and G41S H-ferricyt, respectively. The dependence of the observed rate constant (k_obs_) for N_3_^−^ binding obtained from plots such as those in the inset to Fig. 2A is under the [N_3_^−^] measureable, almost linear (*vide infra*) for both WT and G41S (Fig. 2C). Since the apparent p*K* values for the alkaline transitions of WT and G41S H-ferricyt are different, at 9.3 and 8.2, respectively, N_3_^−^ binding experiments at higher pH values (see Figs S2 and S3) were conducted and revealed that the alkaline transition is not significant for the binding of N_3_^−^ to H-ferricyt at pH 7. It is therefore concluded that N_3_^−^ binding to the G41S variant occurs faster and with a higher binding affinity than it does to WT H-ferricyt.

Ligand binding to the heme iron of ferricyt is generally discussed in terms of an SN1 mechanism as depicted in Fig. 3, where the hexacoordinate ferricyt is in equilibrium with a pentacoordinate form in which the Met80 is dissociated from the heme. This equilibrium lies strongly in favour of the hexacoordinate form, with N_3_^−^ binding to the pentacoordinate form yielding the low-spin hexacoordinate N_3_^−^ adduct (Fig. 3). The mechanism given is amenable to full analysis but this leads to unwieldy equations to describe the kinetic behaviour of the system. However, a simplifying assumption can be made in that the concentration of the pentacoordinate species, that is at all times small, is in a steady-state throughout the reaction^29^. Furthermore, as the [N_3_^−^] is high relative to the [H-ferricyt] the binding kinetics may be considered to be pseudo-first order. These simplifications allow for equations 1 and 2 to be derived that describe the *K*_app_ (Eq. 1) and the dependence of k_obs_ for N_3_^−^binding as a function of [N_3_^−^] (Eq. 2).


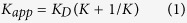



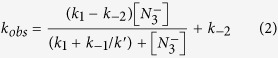


where K = k_1_/k_−1_ and K_D_ = k_−2_/k_2_, with k_2_ the pseudo-first order rate constant for N_3_^−^ binding *i.e.* k_2_ = k’[N_3_^−^], k’ the second order rate constant and k_obs_ is ~k_1_ at high [N_3_^−^]. Equation 1 shows that if K < 1 (as here where the pentacoordinate concentration is very low) then *K*_app_ is ~K_D_/K. Assuming that N_3_^−^ binding to the pentacoordinate species is not directly affected by a distant mutation in the protein, then *K*_D_ will be the same for both proteins and the ratio of *K*_app_ values (^WT^K_app_/^G41S^K_app_) reflect the ratio of K values (^G41S^K/^WT^K). On this basis ^G41S^K > ^WT^K and thus the pentacoordinate form is more populated in the G41S variant than in the WT protein.

Equation 2 predicts that plots of k_obs_ versus [N_3_^−^] follow hyperbolae that intercept the k_obs_ axis at the value of k_−2_, the N_3_^−^ dissociation rate constant, and plateau at k_1_, the Met80 dissociation rate constant. Fitting the data in Fig. 2C to Eq. 2 gives k_−2_ values of 3.5 ± 0.4 and 8.9 ± 0.3 s^−1^ for WT and G41S, respectively. Over the [N_3_^−^] range that was accessible experimentally the hyperbolic curvature was not discerned and thus reliable values of the Met80 dissociation constants (k_1_) cannot be provided. However, it is possible to estimate minimum values for k_1_ of 5.77 ± 1.5 and 45 ± 18 s^−1^, for WT and G41S, respectively, consistent both with Eq. 2 and the data (Fig. 2C). The k_1_ for H-ferricyt may be compared to the value of 11–16 s^−1^ for horse ferricyt obtained by non-linear fitting procedures of the data reported by Sutin and Yandell using N_3_^−^ as the competing ligand^6^. The differences between horse and H-ferricyt is possibly associated with the sequence difference at position 83 in the 71–85 Ω-loop that may effect the dynamics of the Met80 ligand as proposed by Bowler and co-workers^33^. Thus the considerably larger estimates for the rate of Met80 dissociation for the G41S variant is again consistent with the pentacoordinate form being more populated in the G41S variant than in the WT protein, pointing towards a more flexible and labile heme crevice.

### The lowest free energy cyt foldon has increased H/D-exchange in the G41S variant

Monitoring H/D exchange for backbone amide protons with NMR spectroscopy is a tool that provides per residue resolution of structural features and thermodynamic stability of a protein, with exchange rates influenced mainly by hydrogen bonding, particularly in secondary structure, and solvent accessibility^34^. A convenient form of expressing variation in H/D exchange behaviour is to compare the amide protection actors (PF), defined as the ratio between the observed experimental and intrinsic exchange rates (k_ex_/k_in_), where the intrinsic exchange rates are the rates of exchange for the given amino acid sequence, pH and temperature determined using unstructured peptides^35^. Table S1 reports the exchange rates, calculated PFs, free energies of exchange (ΔG_ex_) and proton occupancy values for each residue. For both WT and G41S H-ferricyt, 32 and 46 amides, respectively, are fully exchanged within the first 10 min. Figure 4A shows the sequence dependence of the PFs for the 63 remaining residues of WT for which H/D exchange rates could be calculated and Fig. 4B, the comparable figure for the 44 residues for which H/D exchange data could be determined for the G41S variant. The H/D exchange profile of WT can be broadly divided into six continuous regions of increased protection, residues 6–15, 32–45, 51–54, 64–70, 73–75 and 90–103 (Fig. 4A). With the exception of segment 32–45, the protected regions correlate well with the presence of α-helical secondary structure (Fig. 5A). This profile is largely retained for the G41S variant, however the helix 2 region (residues 51–54) and residues between 27–43 are notable exceptions (Fig. 4B). As is clear from Fig. 5B, many of the residues in the G41S variant having reduced protection to H/D exchange are close to the heme on the Met80 side of the heme. This is consistent with the results of the N_3_^−^ binding experiments, which shows that there is a greater amount of the pentacoordinate form for the G41S variant.

**Figure 1:**
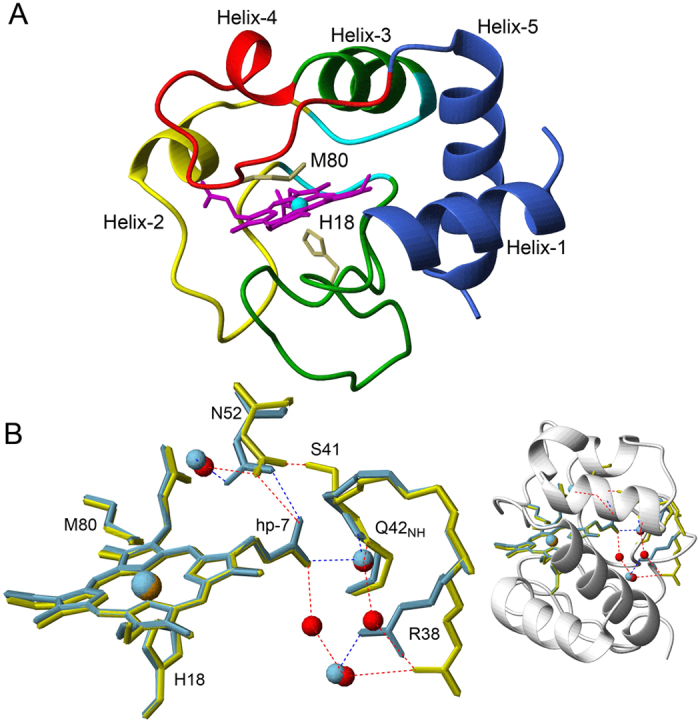
Structure of human cytochrome *c*. (**A**) Cartoon representation of H-cyt with the five foldons identified in horse cyt indicated and coloured as follows: the blue unit (N and C-terminal helices, residues 1–13, 86–104), the green unit (residues 14–36 Ω-loop, 62–70), the cyan neck unit (residues 37–39 and 58–61), the red unit (residues 71–85 Ω-loop) and the yellow unit (residues 40–57 Ω-loop). The His18 and Met80 ligands are labelled (PDB code: 3ZCF)^21^. (**B**) Positional movement of the Arg38 and Asn52 side chains identified in the G41S variant (yellow, PDB code: 3NWV)^20^ compared to the WT protein (light blue, PDB code: 3ZCF)^21^. H-bond networks are illustrated as blue (WT) and red broken lines (G41S), with H_2_O molecules shown as light blue (WT) and red (G41S) spheres. Note that in the G41S structure two additional H_2_O molecules neighbour Arg38.

**Figure 2:**
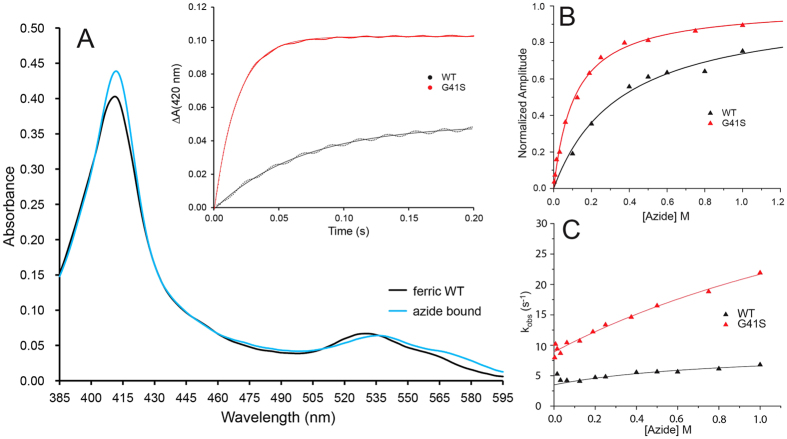
Stopped-flow kinetics of N_3_^−^ binding to H-ferricyt at pH 7 and 25 °C. (**A**) Spectra generated on mixing H-ferricyt with N_3_^−^. Global analysis indicated a single transition with the initial spectrum (black) and the N_3_^−^ bound spectrum (light blue). Inset: time courses for N_3_^−^binding to WT (black) and G41S (red) (5  μM after mixing) are shown together with exponential fits. (**B**) The normalised amplitudes of the time courses recorded at 420 nm plotted against [N_3_^−^]. Binding curves are fitted to a hyperbolic equation to yield the *K*_app_ values reported in the main text. (**C**) The rate constant (k_obs_) determined for N_3_^−^ binding with solid lines representative of fits to Eq. 2, to give the k_1_ and k_-2_ values reported in the main text.

**Figure 3:**
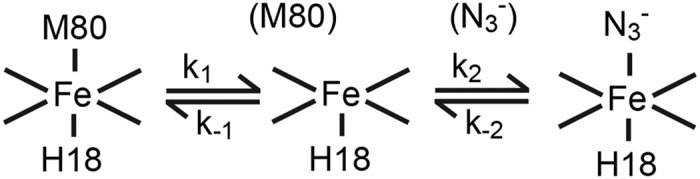
SN1 mechanism of N_3_^−^ binding to H-ferricyt. The dissociation and re-association of the Met80 ligand to the Fe(III) occur with rates of k_1_ and k_−1_, respectively, prior to N_3_^−^ association.

**Figure 4:**
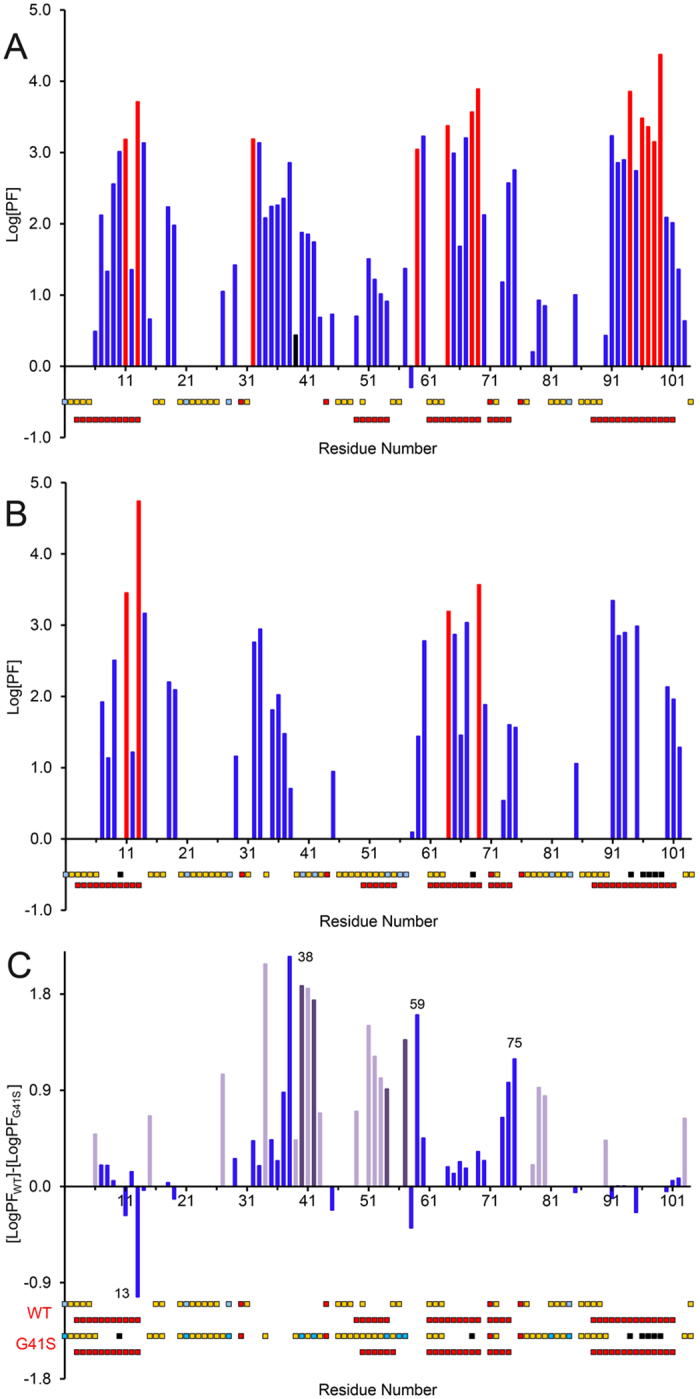
Backbone amide proton H/D exchange analysis of H-ferricyt and its G41S variant. PFs for WT (**A**) and the G41S variant (**B**) plotted against the residue number. For WT, 12 residues underwent fast-exchange (logPF ≤ 1), 16 medium (1 < logPF ≤ 2), 23 slow (2 < logPF) and 12 extremely slow-exchange (residual proton occupancy >80% after ~68 h, corresponding to exchange rates < 0.003 h^−1^, shown in red). For G41S, 4 residue underwent fast-exchange, 14 medium, 15 slow and 4 extremely slow-exchange (residual proton occupancy >80%, after ~72 h, corresponding to exchange rates < 0.003 h^−1^, shown in red). Secondary structure (α-helix) is indicated by the bottom red squares, while red squares above represent Pro residues. Unassigned and very fast-exchanging residues are indicated in light blue and yellow squares, respectively. Residues for which PFs could not be calculated due to the slowness of the exchange in the G41S variant are indicated with black squares (7 residues). (**C**) PF difference plot of [logP_WT_]-[logP_G41S_] shown in blue. Residues that are fully exchanged in G41S have the PFs observed for WT displayed (light purple). Residues that are unassigned only for the G41S variant have the PFs observed for WT displayed (dark purple).

**Figure 5:**
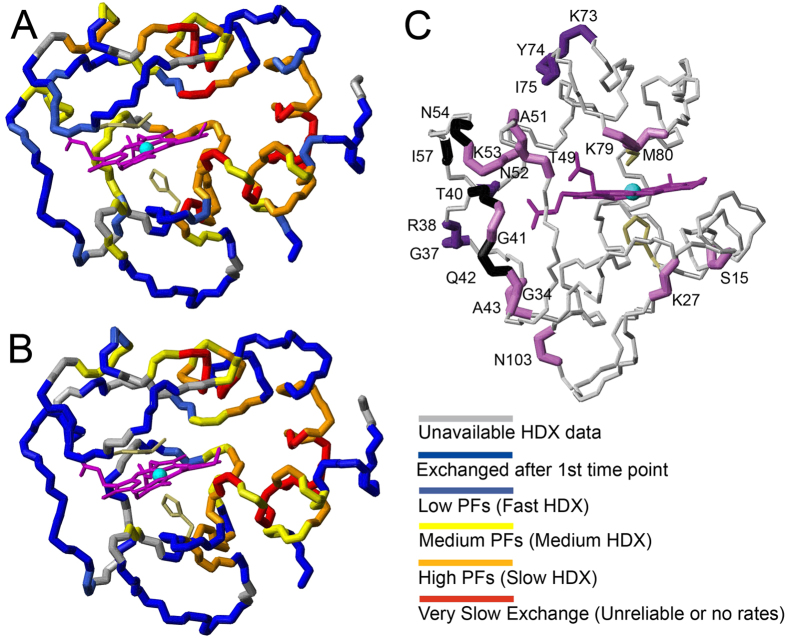
PF mapping for H-ferricyt. PFs are colour coded as indicated on (**A**) the WT and (**B**) the G41S X-ray structures^20^,^21^. The WT cartoon representation in Fig. 1A can be used to aid orientation. (**C**) H/D exchange difference profile mapped onto the X-ray structure of the G41S variant. Residues highlighted in violet have PF > 0.5 in WT but are fully exchanged in G41S, residues highlighted in dark violet have [∆(LogPF_WT_-LogPF_G41S_) > 0.5] and residues highlighted in black have PF > 0.5 in WT but are unassigned for G41S.

The H/D exchange data for H-ferricyt is comparable to that reported for yeast and horse ferricyt^36^,^37^. Protons showing the slowest exchange are mostly confined to the N- and C-terminal foldon^37^,^38^. In general, H/D exchange for amides that are in H-bonds in the ground-state structure of a protein can occur via global unfolding of the protein, through local unfolding, or by a combination of the two. An indicator that global unfolding is the key event is shown by a correspondence between the ΔG_stability_, as determined from denaturant-induced equilibrium unfolding, and ΔG_ex_ for the amide H/D exchange process. When ΔG_stability_ > ΔG_ex_ local unfolding promotes the H/D exchange. This is the case in horse ferricyt where most of the amides involved in H-bonds in the ground-state structure have ΔG_stability _> ΔG_ex_ and thus local unfolding is important for this protein^36^. This is also the case here for WT H-ferricyt as ΔG_stability_ measured under the same conditions as the H/D exchange (Table S2) is significantly greater than the ΔG_ex_ values given in Table S1. Maity *et al*.^39^ describe H/D exchange in horse cyt in terms of five foldon units that fold with different folding free energies in a stepwise sequential manner. The foldon with the greatest stability corresponds to the C-terminal and N-terminal α-helices, whilst the two foldons with the lowest stability are the 71–85 and 40–57 Ω-loops both involving residues packed around the heme (Fig. 1A)^16^,^39^. Our H/D exchange data for H-ferricyt are broadly in agreement with their foldon model (Figs 4 and 5).

While WT and G41S H-ferricyt share a broadly similar H/D exchange profile, differences are apparent (Fig. 4C). The decreased level of protection in the G41S variant maps onto the three lowest stability foldons (Fig. 1A)^39^ and are assigned to residues 34–43 (the neck), 51–54 (40–57 Ω-loop), 59 and 60 (the neck), 73–75, 79 and 80 (71–85 Ω-loop) (Fig. 5C), which predominately encompass the heme propionate-7 region of the protein, whose conformation is altered in the G41S variant (Fig. 1B)^21^. The most affected residues of G41S in terms of increased susceptibility to H/D exchange are Arg38 and Trp59 (the neck), Ile75 (71–85 Ω-loop) and their near neighbours, but Lys27 and Gly34 (14–36 Ω-loop), Ser41, Asp51, Asn52, Lys53 (40–57 Ω-loop), and Lys79 and Met80 (71–85 Ω-loop) also stand out with a significantly diminished H/D exchange protection compared to the corresponding residues of WT (Fig. 4C). Residues Asn52 and Trp59 are conserved in 99% of the mitochondrial cyt sequences and the H-bonds between their side chains and propionate-7 are expected to be conserved in 90% of the same sequences^38^. However, there are more potential H-bonding groups within H-bonding distance of the heme propionates than can be simultaneously accommodated in H-bonds^22^. The apparent differences in H-bond networks in different structures suggests that some of the weaker H-bonds are continually being broken and remade, a view that ties in with the demonstration from H/D exchange that 40–57 Ω-loop has considerable dynamics^16^,^17^. The rapid H/D exchange for the amide resonances of residues Thr40, Gln42, Gly54, Gly56 and Ile57 in the G41S variant is further evidence that this region of the variant has increased dynamics since most of these residues have significant H/D exchange protection in the WT protein.

### The G41S variant has enhanced conformational exchange in all three Ω-loops

**Figure 6:**
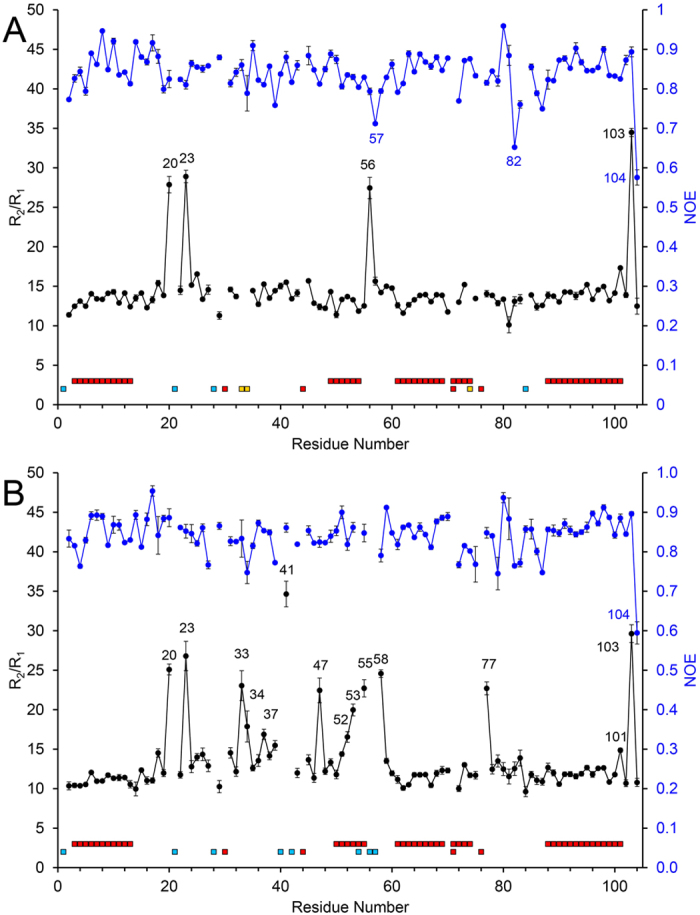
^15^N Relaxation parameters for H-ferricyt. R_2_/R_1_ (in black) and {^1^H}-^15^N NOE (in blue) for WT (**A**) and G41S (**B**). α-helices are indicated by the top red squares while below Pro residues and unassigned residues are indicated with red and light blue squares, respectively. Residues excluded from the analysis due to peak overlap or weak intensity are indicated with yellow squares.

Backbone amide groups of ^15^N-labelled proteins have been extensively used to identify regions of proteins that undergo a range of dynamics on both a ps-ns and μs-ms timescale^24^. Out of the 96 assigned backbone resonances for the WT protein, full relaxation data were obtained for 93 residues (Fig. S4). The average relaxation rates (R_1_ (1/T_1_) and R_2_ (1/T_2_)), for the WT protein were R_1_ = 0.99 ± 0.01 s^−1^ and R_2_ = 14.23 ± 0.22 s^−1^ with an average NOE = 0.84 ± 0.01. Full relaxation data were obtained for all 92 assigned backbone resonances of the G41S variant (Fig. S4). The average values obtained were: R_1_ = 1.07 ± 0.03 s^−1^, R_2_  = 14.48 ± 0.38 s^−1^ and NOE = 0.84 ± 0.01. Heteronuclear {^1^H}-^15^N NOE values are indicators of motions in the ps timescale with values close to 1 suggesting rigidity and lower values indicating increased local flexibility. Both WT and the G41S variant are rigid in this respect with only the C-terminal residue E104 having a NOE value below 0.65 (Fig. 6A,B). The R_1_ rates are sensitive to motion on the ns to ps timescales, and are relatively constant throughout both the WT and G41S sequence. However, a handful of R_2_ rates for the WT are significantly increased above the average, notably those for residues Val20, Gly23, Gly56 and Asn103, probably reflecting contributions to relaxation from slower conformational exchange contributions, as observed with other proteins (Fig. S4). Significantly more residues for the G41S variant have R_2_ rates deviating from the average (Fig. S4). Plots of R_2_/R_1_ ratios, which are a useful indicator for identifying residues experiencing chemical exchange, show that the G41S variant has significantly more residues displaying slower conformational exchange behaviour than the WT protein (Fig. 6A,C). In particular, residues in the 19–35 Ω-loop (Val20, Gly23, His33, Gly34), the neck unit (Gly37, Arg38, Lys39 and Ile58), the 40–57 Ω-loop (Ser41, Ser47, Ala51, Asn52, Lys53, Lys55), the 71–85 Ω-loop (Gly77) and A101 and N103 at the end of the C-terminal α-helix.

**Figure 7:**
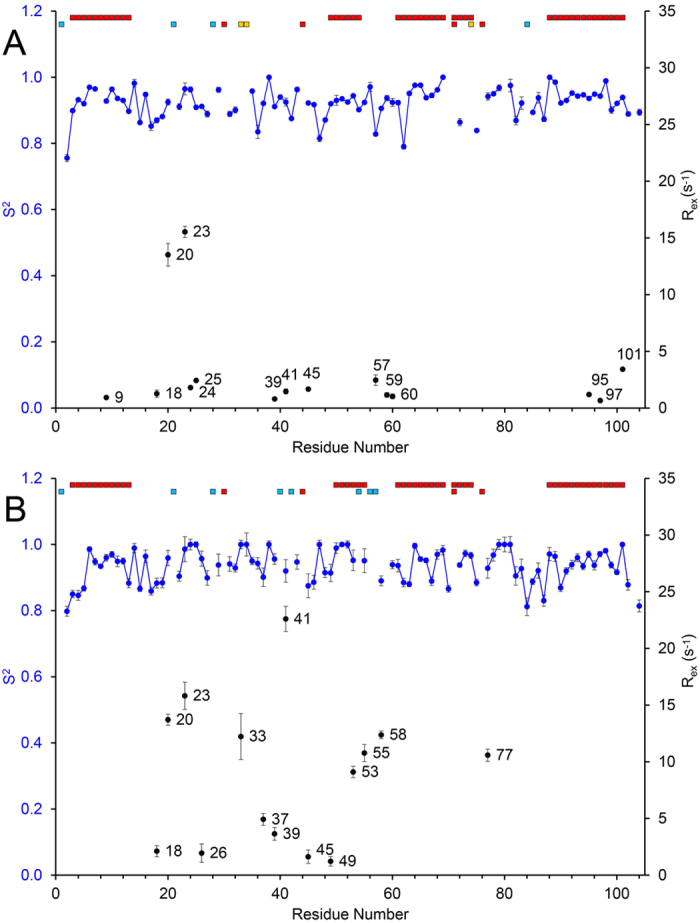
Model-free parameters for H-ferricyt. S^2^ order parameters (in blue) and R_ex_ contributions (in black) for WT (**A**) and G41S (**B**). α-helices are indicated by the top red squares while below Pro residues and unassigned residues are indicated with red and light blue squares, respectively. Residues excluded from the analysis due to peak overlap or weak intensity are indicated with yellow squares.

Further analysis of the relaxation data was performed with the Model-free formalism^40^ (see SI and Table S3), which provides three main dynamic parameters: a) the generalized order parameter, S^2^, b) the effective correlation time, τ_e_ and c) the chemical exchange contribution rate, R_ex_. The average S^2^ values, which specify the degree of spatial restriction of the N-H bond, for WT and the G41S variant are comparable at 0.924 ± 0.046 and 0.934 ± 0.050, respectively (Fig. 7). This is consistent with both proteins being relatively rigid, presumably due to the α-helical secondary structure and the compact core held together by hydrophobic interactions associated with the heme. For WT H-ferricyt, large R_ex_ terms (between 12 and 16 s^−1^) were observed for Val20 and Gly23 (average of 14.52 ± 1.01 s^−1^) and smaller R_ex_ terms (<5 s^−1^) for residues Ile9, His18, Gly24, Lys25, Lys39, Gly41, Gly45, Ile57, Trp59, Gly60, Ile95, Tyr97 and Ala101 (average of 1.56 ± 0.76 s^−1^) (Fig. 7A). For the G41S variant, large R_ex_ terms (between 8 and 16 s^−1^) were observed for Val20, Gly23, His33, Ser41, Lys53, Lys55, Ile58 and Gly77 (average of 13.40 ± 3.97 s^−1^) and smaller R_ex_ terms (<5 s^−1^) for His18, His26, Gly37, Lys39, Gly45 and Thr49 (average of 2.58 ± 1.29 s^−1^) (Fig. 7B). Overall, 13 residues have R_ex_ terms of small magnitude and 2 residues large magnitude for WT, while for the G41S variant 6 residues have R_ex_ terms of small magnitude and 8 residues large magnitude, thus indicating enhanced conformational exchange on the ms to μs timescale in the G41S variant. The less informative number of residues with a correlation time (τ_e_) indicating internal motion was 23 and 20 for the WT and G41S variant, respectively (Fig. S5), with many of these assigned to model 5, which reflects both fast and slow motions (Table S3) and is difficult to accurately characterise with relaxation data. The significant finding is the number of residues exhibiting relatively large R_ex_ terms.

**Figure 8:**
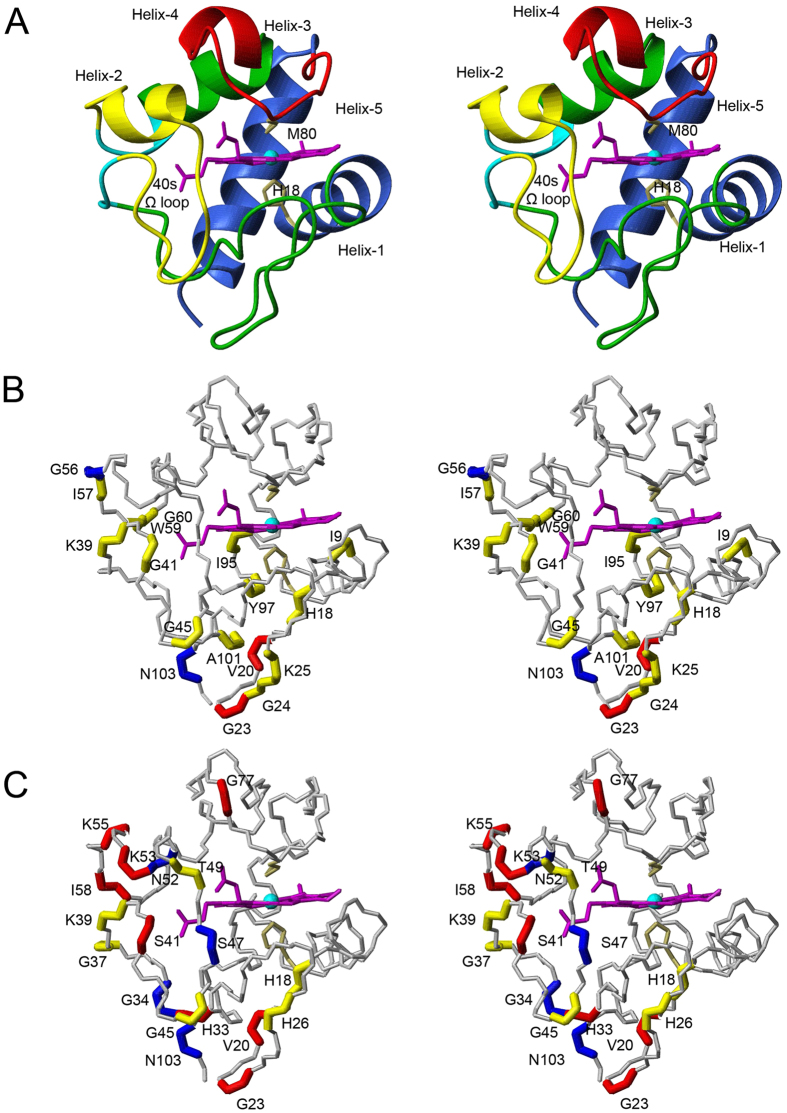
Dynamic profile mapping of H-ferricyt. Stereo pairs representation of (**A**) WT with the five foldon units coloured as described in the legend to Fig. 1A. NMR dynamics plotted on the X-ray structures of WT (**B**) and G41S (**C**). Residues highlighted in yellow and red have low (<5 s^−1^) and high (>5 s^−1^) R_ex_ terms, respectively, with those in blue having R_2_/R_1_ deviating from the average but without an associated R_ex_ term. In all depictions heme and axial ligands are shown in stick representation.

The distribution of residues requiring an R_ex_ term to describe their relaxation data for both WT (15 residues) and G41S (14 residues) H-ferricyt have been mapped onto the respective structures in Fig. 8. A striking overall resemblance to the H/D exchange difference map shown in Fig. 5C is observed. Furthermore it highlights that under native conditions the regions experiencing conformational exchange for both the WT and G41S proteins are essentially identical, with the switching of a glycine to a serine at position 41 enhancing the dynamics in 3 out of the 5 H-ferricyt foldons, and in particular the lowest energy foldon (40–57 Ω-loop). Thus the G41S variant nicely illustrates a previous prediction that foldon substructure may determine additional properties^16^, such as apoptotic activity.

### Conformational dynamics in the 40–57 Ω-loop modulate the pentacoordinate form and apoptotic interactions

The NMR data and N_3_^−^ binding kinetics strongly support a linkage in the dynamics of the region surrounding heme propionate-7, *i.e.* the 40–57 Ω-loop and lability of the Met80 heme ligand. A recent X-ray structure of the K72A variant of yeast cyt provides further compelling evidence of a direct conformational link between Met80 and the heme propionate-7 region by being a conformer in which the Met80 ligand is dissociated from the heme and the Arg38 side chain has moved in a similar manner to that observed in the G41S structure (Fig. 1B)^20^,^33^. These structural changes coincide with the creation of a H_2_O channel leading from the vacant Met80 position to beyond heme propionate-7 and ending at the Arg38 side chain, resembling more the conformation of the G41S H-ferricyt than the WT protein in this region. This channel, with Arg38 proposed to act as a seal, has been suggested to enable facile entry of H_2_O_2_ into the heme environment and/or provide a H^+^ exit route^33^. β-sheet formation in the 40s region of the 40–57 Ω-loop has been reported for yeast ferricyt at low pH as a result of breaking a H-bond between His26-Pro44. This was suggested to trigger rearrangement in the Met80 loop since β-sheet formation was accompanied by dissociation of Met80 from the heme iron^41^,^42^. This event also causes disruption of the H-bonding network associated with heme propionate-7 resulting in a change of the heme position relative to the polypeptide and increased mobility for aromatic residues such as Trp59^41^,^42^. A further recent crystal structure of the T78C/K79G yeast cyt variant reveals a non-native form whereby Lys73 replaces the Met80 ligand and a conserved stretch of the Met80 loop (^76^PGTK^79^) refolds into a β-hairpin structure, with Gly77 being a critical element of this hairpin and central to the flexibility of this peptide fragment^43^. Furthermore, a positional change in helix-2 (residues 51–54) occurs to accommodate the refolded Met80 loop which has the effect of increasing the volume of the heme pocket and causing peroxidase activity to increase in this T78C/K79G variant^43^. Facets of these structural changes in this non-native conformer are therefore comparable with the regions displaying increased dynamics in the G41S variant. This suggests that, in solution, alternative conformations are kinetically and thermodynamically accessible in the G41S variant, whereby the heightened dynamics in 40–57 Ω-loop foldon is coupled to an increased population of a peroxidase pentacoodinate state, resulting in the elevated peroxidase activity akin to the WT protein when bound to CL^18^,^19^. In addition to the X-ray structures of the variants mentioned above, an important finding from H/D exchange studies is that the dynamics of the 40–57 Ω-loop controls the rate of the alkaline isomerisation of ferricyt, which leads to Met80 being replaced by a lysine as an axial ligand^44^. Though the alkaline isomerisation is not a factor in our N_3_^−^ binding experiments, and thus not a factor in generation of the pentacoordinate, it is a further demonstration that the dynamics of the 40–57 Ω-loop and Met80 lability are linked and can govern function^16^.

Finally, the 40–57 Ω-loop is the least conserved in cyt evolution. This has been highlighted recently through the discovery of a species-dependent interaction between cyt and Apaf-1 in cytosolic extracts, which is proposed to arise due to sequence variation between mitochondrial species in the 40–57 Ω-loop^45^. Further to this discovery, residues flanking Pro44 have for some time been associated with a conformational change in cells undergoing apoptosis^46^ and recent modelling studies suggest H-cyt interacts with Apaf-1 through contact via the 40–57 Ω-loop^45^,^47^. Thus based on the present work we propose that the dynamics of 40–57 Ω-loop may be species-specific and tuned to regulate interaction with the cognate Apaf-1.

## Experimental

### NMR sample preparation

^15^N-labelled samples of WT H-ferricyt and the G41S variant ranging in concentration from 0.8 to 1 mM were used for all experiments and prepared as described previously^48^.

### NMR Spectroscopy

All NMR experiments were performed at 288 K (unless specified otherwise) and acquired on a Bruker 800 MHz spectrometer equipped with either a 5 mm HCN inverse triple resonance z-axis gradient probe or a 5 mm broad band inverse z-axis gradient probe, and a Bruker 500 MHz spectrometer equipped with a 5 mm HCN inverse triple resonance z-axis gradient probe. Data were processed with either Topspin (Bruker Biospin) or the NMRPipe and NMRDraw package^49^. Data analysis was performed with the programs CCPNmr Analysis^50^ and Sparky^51^. The amide chemical shifts used in this work for the WT and G41S variant have been previously determined (BMRB accession numbers 25418 and 25422, respectively)^48^.

### H/D exchange

Freeze-dried ^15^N-labelled and desalted samples of both WT and G41S H-ferricyt at 1 mM concentration were re-dissolved in 20 mM sodium phosphate pH 6.5, 50 mM NaCl pre-made in 99.9% ^2^H_2_O (Sigma). All H/D exchange NMR data were acquired at 500 MHz as matrices of 1024 × 64 complex data points with 4 scans and spectral widths of 15 (^1^H) and 42 (^15^N) ppm (39.8 ppm for G41S) centred at 4.7 and 118.6 ppm, respectively. The acquisition of consecutive ^1^H-^15^N-HSQC spectra started approximately 5 min after sample resuspension. Acquisition proceeded for WT with consecutive HSQC spectra of approximately 5 min acquisition time for a period of 68 h, totalling 792 experiments. The first 49 experiments (to ensure maximum coverage of the fast-exchanging residues) were followed by 74 experiments at 50 min intervals, totalling 123 experiments, which were used for the extrapolation of the H/D exchange rates. For the G41S variant, acquisition of ^1^H-^15^N-HSQC spectra of identical acquisition parameters was executed at specific timed intervals, *i.e.* 49 consecutive experiments followed by 79 experiments at 50 min intervals. CCPNmr Analysis was used to fit peak volumes as a function of time to a two-parameter single exponential decay function^50^. Rates of exchange were extracted from this fitting and were used for the calculation of PFs^35^,^52^. Intrinsic exchange rate constants required for the calculation of PFs were obtained as described previously^35^. ∆G_ex_ (Table S1) values were obtained from ∆G_ex _= -RTln(PF) where R is the gas constant and T is the temperature in Kelvin^52^.

### ^15^N relaxation data

^15^N T_1_, T_2_, and {^1^H}-^15^N NOE data were collected at 800 MHz. Spectra were acquired as matrices of 1024 × 256 complex data points with 8 scans for T_1_ and T_2_ data and 64 scans for {^1^H}-^15^N NOE data. Spectral widths of 15 (^1^H) and 39.3 (^15^N) ppm centred at 4.7 and 118.6 ppm respectively, were used. T_1_ experiments were acquired with relaxation delays of 10, 50, 80, 200, 500, 750, 2000 and 3500 ms with duplicates at 10, 200 and 500 ms. T_2_ experiments were acquired with relaxation delays of 16.96, 33.92, 50.88, 67.84, 101.76, 135.68, 203.52 and 254.40 ms with duplicates at 16.96, 50.88 and 101.76 ms. Recycle delays for both experiments were 5 s. The repeated relaxation delays were used for the determination of peak height uncertainties^53^. Identical delays were used for both WT and G41S with the exception of the first T_1_ delay for G41S, which was 20 ms due to pulse sequence limitations. Both T_1_ and T_2_ series of experiments were acquired with interleaved delay values to minimize sample heating. T_1_ and T_2_ data for G41S were acquired as a pseudo-3D experiment, and processed as individual 2D planes using NMRPipe^49^. T_1_ and T_2_ values were extracted through the fitting of peak heights as a function of the relaxation delay to a two-parameter single exponential decay function using the follow intensity changes function of CCPN Analysis. These were subsequently converted to R_1_ and R_2_ rate constants (R, where R_1_ = 1/T_1_ and R_2_  = 1/T_2_). The fit errors for T_1_ and T_2_ were also converted into R_1_ and R_2_ errors through the equality of the T_1_/T_2_ and R_2_/R_1_ errors. Heteronuclear {^1^H}-^15^N NOEs were measured with a proton saturation period of 5 s. Interleaved saturated and unsaturated experiments were acquired in triplicate in order to determine the experimental errors as the standard deviation of the average NOE value. NOE values were calculated as the ratio of the peak heights with and without proton saturation.

### Model-free analysis

Model-free analysis was performed using the Model-free4 program^54^ complemented with the FAST-Modelfree program^55^ to facilitate and speed-up data analysis and interpretation. The first step consisted of the estimation of the rotational correlation time using the program r2r1_tm (http://www.palmer.hs.columbia.edu/) from the relaxation data. The program quadric_diffusion (http://www.palmer.hs.columbia.edu/) was used to calculate the rotational diffusion parameters from the output local correlation times. This program uses a 3D structure translationally centred on the centre of mass and rotated to the inertia tensor principal axes. These manipulations were achieved with the program pdbinertia (http://www.palmer.hs.columbia.edu/). Initial pdb manipulations preceding the use of pdbinertia were performed with molecular graphics program MOLMOL^56^. Quadric_diffusion also produces a pdb structure rotated to axially symmetric principal axes, subsequently used as an input for FAST-Model-free. The paramagnetic contribution from the low-spin (S = ½) heme to the nitrogen relaxation rates is considered negligible because of the low gyromagnetic ratio of the ^15^N nucleus^57^. The Tjandra-Bax conditions^58^ were applied in order to exclude residues with a large-amplitude fast internal motions and residues subject to conformational exchange from the rotational correlation time calculations. The former applies if the {^1^H}-^15^N NOE < 0.65 and the later applies if [(T_2,av _− T_2,n_)/T_2,av _− (T_1,av _− T_1,n_)/T_2av_] > 1.5 x standard deviation, where T_1,av_ and T_2,av_ the average values, and T_1,n_ and T_2,n_ are the relaxation times for each individual residue, n. These conditions identify the ‘rigid’ residues with relaxation parameter values corresponding to the global rotational dynamics.

The five models considered for the interpretation of dynamic parameters using the Model-free approach^40^,^59^,^60^ were: model 1 (with only the generalized order parameter S^2^), model 2 (with S^2^ and the correlation time for internal motion τ_e_), model 3 (S^2^ and the exchange contribution to the relaxation rate, R_ex_, or model 1 with R_ex_), model 4 (S^2^, τ_e_ and R_ex_ or model 2 with R_ex_) and finally the extended model 5 (with the generalized order parameter for faster and slower timescales, S^2^ = S^2^_f_S^2^_s_ and τ_e_) which accounts for a fast and a slow internal motion. The models with R_ex_ terms (3 and 4) reflect on conformational exchange processes on the ms to μs timescale. The models with effective correlation time reflect on internal motions on the ps to ns timescale. Model-free analysis was performed with ^15^N gyromagnetic ratio of −2.71, N-H bond distance of 1.02 Å and ^15^N chemical shift anisotropy of −160. FAST-Model-free calculations were performed with confidence limit for χ^2^-testing of 0.95, confidence limit for F-testing of 0.80, 50 iterations, sum squared error cut-off of 200, 500–1000 Monte-Carlo simulations and optimization of the diffusion tensor.

### Stopped-flow kinetics

Kinetic experiments were carried out using an Applied Photophysics (Leatherhead, UK) SX20 stopped-flow spectrophotometer equipped with both photomultiplier and diode array detection systems and thermostatted at 25 °C. Stocks of sodium azide 2 M were prepared in 50 mM sodium phosphate and 50 mM MES pH 7 and diluted to the desired N^3−^ concentration with the same buffer but containing 2 M NaCl to maintain the ionic strength. Time courses were taken at 420 nm with known [N^3−^] (between 0.08–2 M before mixing) and 10 μM protein (before mixing) with the transient fitted to a single exponential function yielding both pseudo first-order rate constants and amplitudes. All errors reported are standard errors.

## Additional Information

**How to cite this article**: Karsisiotis, A. I. *et al*. Increased dynamics in the 40–57 Ω-loop of the G41S variant of human cytochrome *c* promote its pro-apoptotic conformation. *Sci. Rep.* **6**, 30447; doi: 10.1038/srep30447 (2016).

## Supplementary Material

Supplementary Information
