# Supplementary material for: Increased dynamics in the 40–57 Ω-loop of the G41S variant of human cytochrome c promote its pro-apoptotic conformation
Source: Sci Rep. 2016 Jul 27;6:30447. doi: 10.1038/srep30447 (PMC4962053; doi:10.1038/srep30447)
Supplement: Supplementary Information [file srep30447-s1.pdf]

Supporting Information

Increased dynamics in the 40-57 Ω-loop of the G41S variant of human cytochrome *c* promote its pro-apoptotic conformation

Andreas Ioannis Karsisiotis[1], Oliver M. Deacon[1], Michael T. Wilson[1], Colin Macdonald[2], Tharin M.A. Blumenschein[2], Geoffrey R. Moore[2], Jonathan A.R. Worrall[1]

[1]School of Biological Sciences, University of Essex, Wivenhoe Park, Colchester, CO4 3SQ, U.K.
[2]School of Chemistry, University of East Anglia, Norwich Research Park, NR4 7TJ, U.K.

RESULTS

Dissolution of lyophilised H-ferricyt

Lyophilised horse heart ferricyt has been reported to exhibit a change in electronic spectrum upon re-dissolving in buffer [1]. We confirmed that H-ferricyt generated the native protein in solution upon dissolution in buffer instantaneously through the measurement of UV-vis spectra, and through comparison of ^{15}N-HSQC spectra of H-ferricyt that had not been subjected to lyophilisation.

N_3^- binding to WT and G41S H-ferricyt at alkaline pH

In order to ascertain the influence of the presence of any alkaline form on N_3^- binding stopped–flow experiments were performed at higher pH values, under which conditions the concentration of the alkaline conformer(s) is (are) increased. The optical transitions following mixing of H-ferricyt with N_3^- revealed that the alkaline forms reacted significantly more slowly than the native form. Moreover, the rate constant for binding to the alkaline form was found to be close in value to that of the conversion of the Lys-bound H-ferricyt to the native Met80 bound form as revealed by independent pH jump experiments. For example (**Fig. S2**), at pH 9, N_3^- (1 M) binding to G41S H-ferricyt was characterised by a small fast phase followed by a large slow phase with rate constants of 19 s^{-1} and 0.086 s^{-1}, respectively. The proportions of these phases as revealed by their amplitudes conformed to expectations for binding to the neutral and alkaline forms, respectively. The rate constant for the faster phase was [N_3^-] dependent, whereas the slower rate constant was not. A similar pattern of results was obtained for WT H-ferricyt with rate constants of 6.1 s^{-1} and 0.085 s^{-1}, respectively. The interconversion of conformers of G41S H-ferricyt was further investigated by a pH-jump experiment in which the pH of a solution was rapidly changed from pH 7 to pH 9 when an optical transition was observed (**Fig. S3**), consistent with ligand exchange between Met80 and Lys and comprising a single exponential with a rate constant of 0.167 s^{-1}, close to the value of 0.085 s^{-1} for N_3^- binding to the alkaline form of the protein (see above), but slightly higher due to a small

term involving the back reaction at this pH [2]. Because the rates of the processes involving the alkaline conformer(s) are so slow, and also because they are independent of N_3^- binding, we conclude that they are not significant for the binding of N_3^- to H-ferricyt under the conditions of Fig. 2 at pH 7.

Model free analysis

The application of the Tjandra-Bax conditions to the data for the WT protein [3] led to the exclusion of E104 from the calculation of the overall correlation time (τ_m) estimate due to the lower than 0.65 NOE value. Residues 20, 23, 56 and 103 were excluded because they have elevated R_2/R_1 ratios. Thus 89 residues were used for the calculation of rotational diffusion parameters from relaxation data. For G41S H-ferricyt, the C-terminal residue E104 was again excluded from the calculation of the overall τ_m estimate due to its low NOE value. Residues 20, 23, 33, 41, 47, 53, 55, 58, 77 and 103 were excluded as they satisfied the conformational exchange criterion. Consequently, 81 residues were used for the calculation of rotational diffusion parameters from relaxation data.

H-ferricyt is an axially symmetric ellipsoid with approximate molecular dimensions of 34 x 30 x 24 Å. The principal components of the inertia tensors were determined using pdbinertia and are:, D_{zz} = 1.0, D_{xx} = 0.8535, D_{yy} = 0.7013 for WT H-ferricyt and D_{zz} = 1.0, D_{xx} = 0.8474, D_{yy} = 0.7061 for G41S H-ferricyt. In both cases there is significant deviation from the spherical (isotropic) tensor. Relaxation data for both WT and G41S H-ferricyt were best treated with tan axially symmetric model having $D_{||} = D_{zz} = 1.0$ and $D_{\perp} = 0.5(D_{xx} + D_{yy}) = 0.7774$ or 0.77675 for WT and G41S H-ferricyt, respectively. The diffusion anisotropy $D_{||}/D_{\perp}$ for the axially symmetric tensor is 1.286 and 1.287 for WT and G41S H-ferricyt, respectively. The program quadric_diffusion further verified the axially symmetric tensor and provided local effective correlation times and tensor parameters based on relaxation data (R_2/R_1 ratios through program r2r1_tm). The average τ_m values used for the calculation of the rotational diffusion parameters for the WT H-ferricyt and its G41S variant were 8.57 ± 0.41 ns and 8.04 ± 0.56 ns, respectively. The diffusion anisotropy and Euler angles obtained through this approach were $D_{||}/D_{\perp}$ = 0.88013, Θ = 1.30242 °, Φ = 3.00910 ° and $D_{||}/D_{\perp}$ = 0.87207, Θ = 0.89751 °, Φ = 0.34915 ° for WT and G41S H-ferricyt, respectively. These values were used as the input of the FASTmodelfree calculations. Discrepancies between the value of $D_{||}/D_{\perp}$ calculated from the structures (1.286 and 1.287) and the relaxation data (0.88013 and 0.87207) have been previously reported [4] and it is likely to be due to the differentiation between prolate and oblate approximations manifested by two minima in conformational space [5]. The

differences in D||/D⊥ values do not lead to significant differences in the identification of residues with chemical exchange contributions to their relaxation rates.

The spin relaxation data for WT and G41S H-ferricyt were best fitted with an axially symmetric rotational diffusion tensor having optimized diffusion anisotropy and Euler angle values of D||/⊥ = 0.936, Φ = -23.189 °, Θ = -8.760 ° and a rotational correlation time (τ_m) of 8.601 for WT H-ferricyt, and D||/⊥ = 0.875, Φ = 99.689 °, Θ = 11.8 ° and τ_m of 7.994 ns for G41S H-ferricyt. A summary of the FASTmodelfree results obtained for WT (and G41S) H-ferricyt residues 8, 70, 73, 80 and 103 (59 and 103) were not assigned to any model, while 88 out 93 residues (88 out of 90) were assigned to a specific model. For models 1 to 5, the distribution of residues was 55 (58), 10 (16), 10 (12), 5 (2) and 8 (2), respectively

REFERENCES

1 Aviram, I. & Schejter, A. On the conformation change attending the hydration of lyophilized cytochrome c. *Biopolymers* **11**, 2141-2145, doi:10.1002/bip.1972.360111014 (1972).

2 Davis, L. A., Schejter, A. & Hess, G. P. Alkaline isomerization of oxidized cytochrome c. Equilibrium and kinetic measurements. *J Biol Chem* **249**, 2624-2632 (1974).

3 Tjandra, N., Feller, S. E., Pastor, R. W. & Bax, A. Rotational diffusion anisotropy of human ubiquitin from N-15 NMR relaxation. *Journal of the American Chemical Society* **117**, 12562-12566, doi:10.1021/ja00155a020 (1995).

4 Sahu, S. C., Bhuyan, A. K., Majumdar, A. & Udgaonkar, J. B. Backbone dynamics of barstar: a (15)N NMR relaxation study. *Proteins* **41**, 460-474 (2000).

5 Blackledge, M., Cordier, F., Dosset, P. & Marion, D. Precision and uncertainty in the characterization of anisotropic rotational diffusion by N-15 relaxation. *Journal of the American Chemical Society* **120**, 4538-4539, doi:10.1021/ja9742646 (1998).

6 Rajagopal, B. S. *et al.* The hydrogen-peroxide-induced radical behaviour in human cytochrome c-phospholipid complexes: implications for the enhanced pro-apoptotic activity of the G41S mutant. *Biochem J* **456**, 441-452, doi:10.1042/bj20130758 (2013).

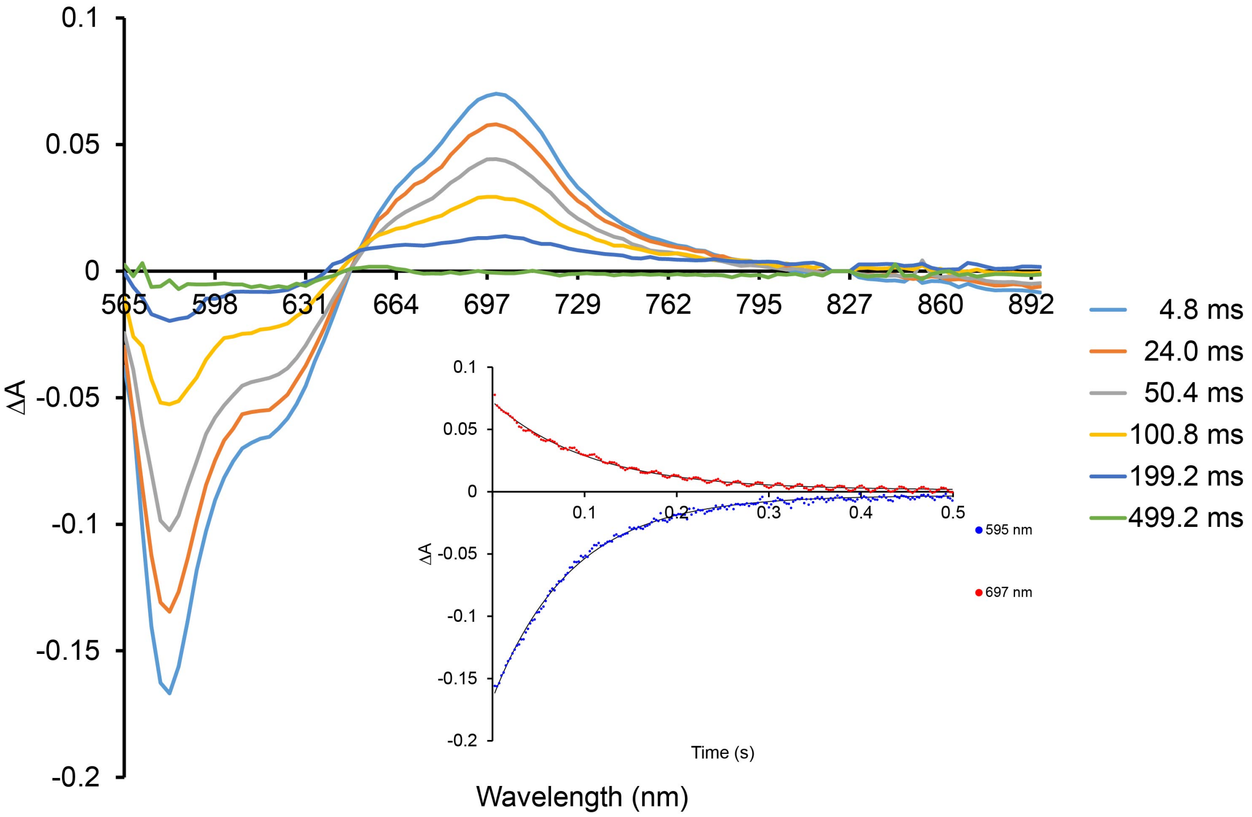

Figure S1: N^{3-} binding to G41S H-ferricyt at pH 7 and 25 ^{o}C. Cyt (425 μM after mixing) was mixed with 0.5 M N^{3-} (after mixing) and difference spectra recorded from 565 to 895 nm. Bleaching of the 695 nm band is observed. The inset depicts time courses and their fits to single exponential at 697 nm (descending) and 595 (ascending).

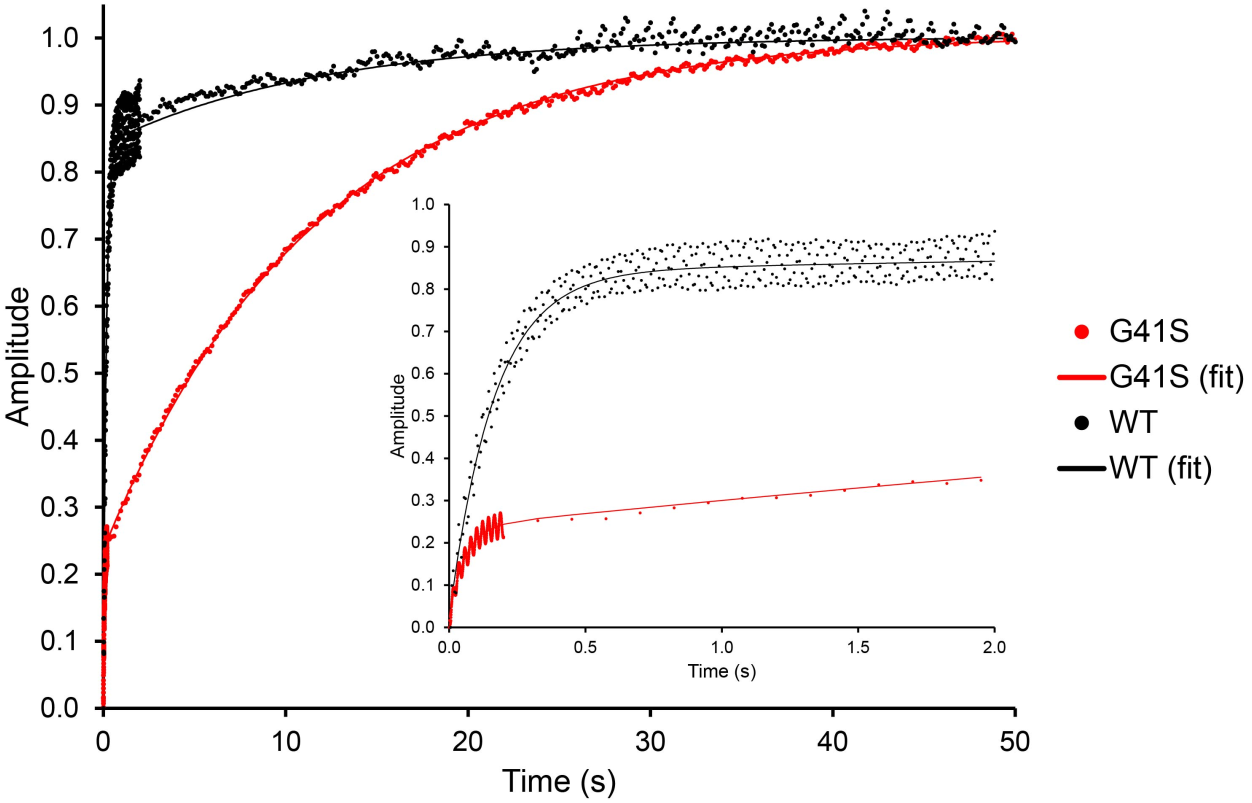

Figure S2: N^{3-} binding to WT and G41S H-ferricyt at pH 9 and 25 ^{o}C. Cyt (5 μM after mixing) was mixed with 1 M N^{3-} (after mixing) and the time courses at 416 nm recorded. The inset shows an expanded view of the first two sec of the time courses.

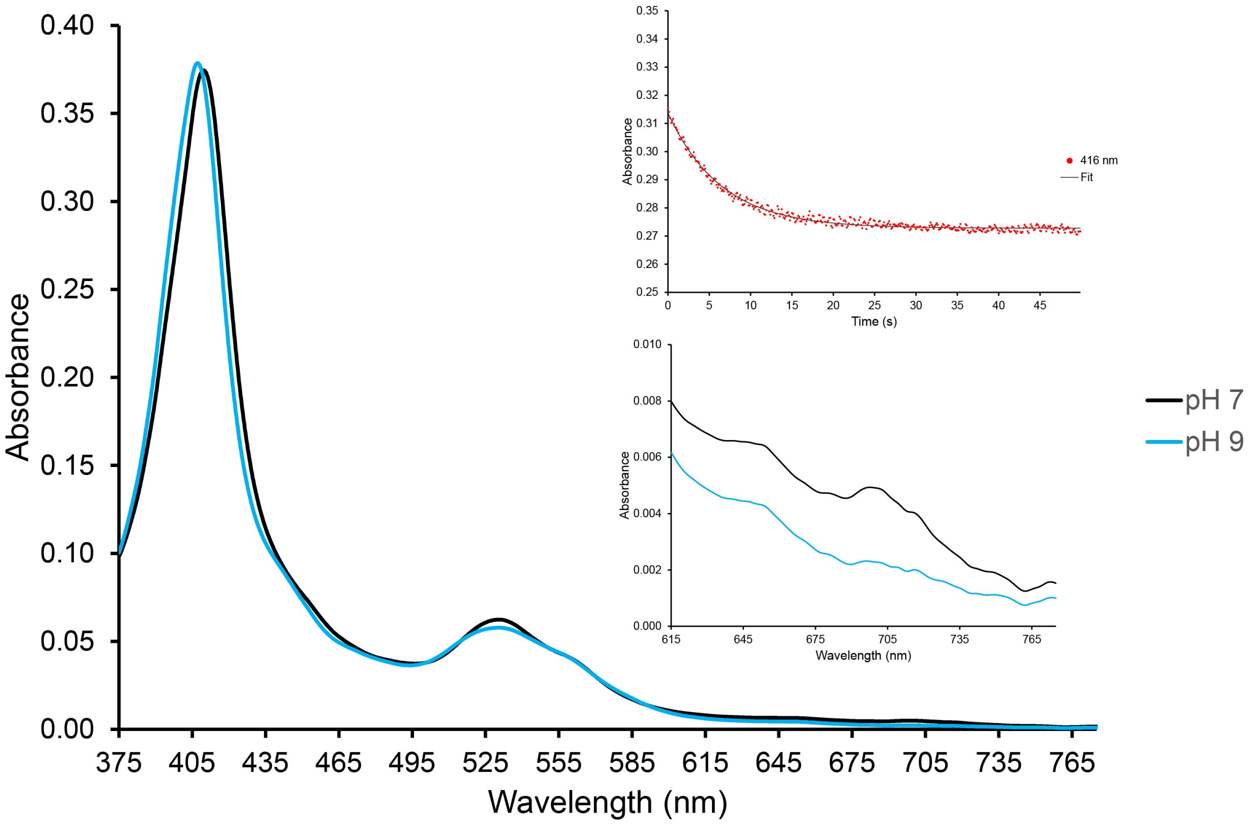

Figure S3: Stopped-flow analysis of pH jump (pH 7 to pH 9) of H-ferricyt at 25 ^{o}C. Spectra displayed were generated by global analysis of the full data set and depict the initial spectrum at pH 7 and the final spectrum at pH 9. Top inset illustrates a time course at 416 nm, fitted to a single exponential. Bottom inset shows an expanded spectrum illustrating the bleaching of the 695 nm band at pH 9. The protein (10 μM) was prepared in 2 mM sodium phosphate, pH 7 and was mixed with 20 mM borate buffer, 100 mM NaCl giving a final pH of 9.

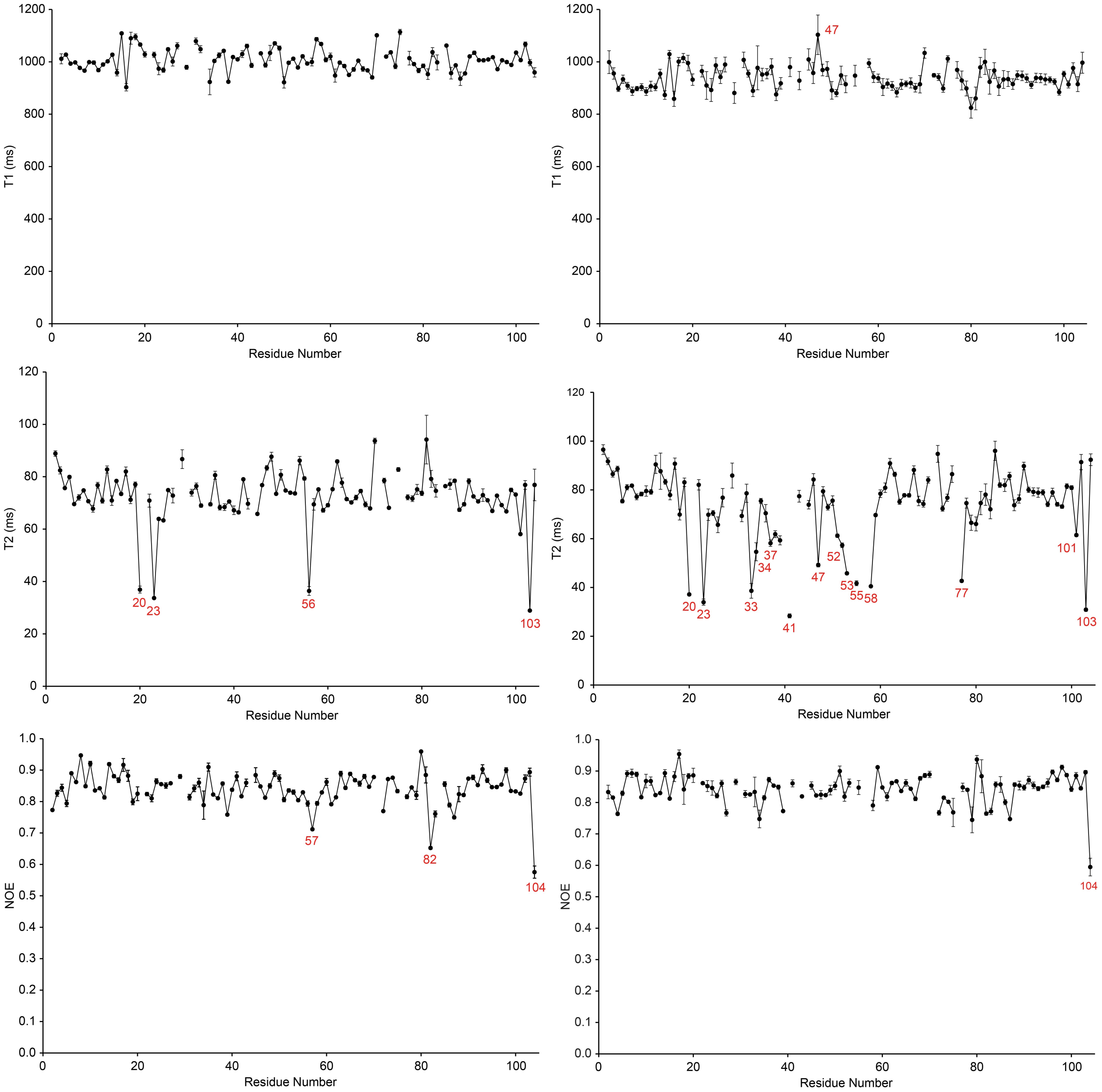

Figure S4: ^{15}N Relaxation parameters for H-ferricyt WT (left) and the G41S variant (right) plotted against the residue number (pH 6.5, 15 ^{o}C).

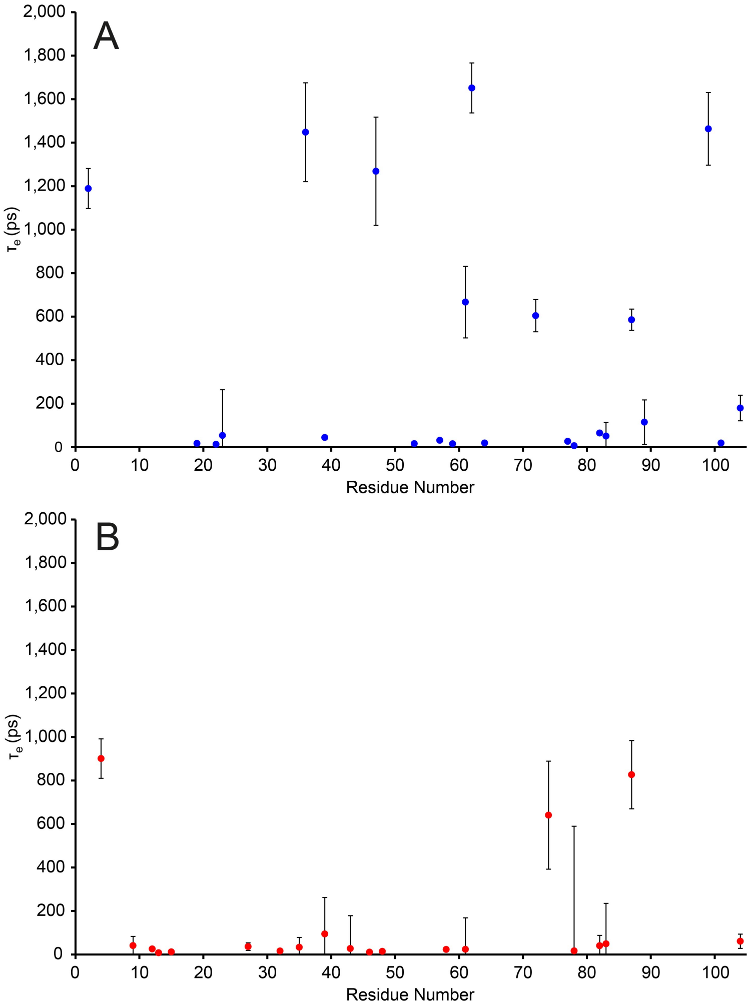

Figure S5: Model free parameters for H-ferricyt WT and the G41S variant (pH 6.5, 15 ^{o}C). Correlation time (τ_e) indicating internal motion for WT (A) and G41S (B).

Table S1: H/D exchange rates, calculated protection factors (log P), free energy of exchange (ΔG_{ex}) and proton occupancy values (Occ) for WT and G41S H-ferricyt (pH 6.5 and 15 ^{o}C). Numbers in red indicate negative k_{ex} values, with the proton occupancy values for these points 1.00 or over.

	WT				G41S			
**Res.**	**k_{ex} (h^{-1})**	**logP**	**ΔG_{ex} (kcal/mol)**	**Occ. (V_i/V_f)**	**k_{ex} (h^{-1})**	**logP**	**ΔG_{HX} (kcal/mol)**	**Occ. (V_i/V_f)**
**G1**	-	-	-	-	-	-	-	-
**D2**	-	-	-	-	-	-	-	-
**V3**	-	-	-	-	-	-	-	-
**E4**	-	-	-	-	-	-	-	-
**K5**	-	-	-	-	-	-	-	-
**G6**	3.52E+00	0.49	0.64	0.22	-	-	-	-
**K7**	5.75E-02	2.12	2.79	0.08	9.07E-02	1.92	2.53	0.14
**K8**	3.29E-01	1.33	1.75	0.06	5.15E-01	1.13	1.50	0.15
**I9**	7.50E-03	2.56	3.37	0.61	8.46E-03	2.50	3.30	0.57
**F10**	3.21E-03	3.01	3.97	0.79	-4.91E-04	-	-	1.09
**I11**	1.64E-03	3.18	4.19	0.92	8.82E-04	3.45	4.55	0.97
**M12**	2.00E-01	1.35	1.78	0.26	2.74E-01	1.22	1.60	0.16
**K13**	1.36E-03	3.71	4.89	0.92	1.27E-04	4.74	6.24	1.00
**C14**	1.30E-02	3.13	4.13	0.47	1.20E-02	3.16	4.17	0.46
**S15**	4.93E+00	0.66	0.87	0.42	-	-	-	-
**Q16**	-	-	-	-	-	-	-	-
**C17**	-	-	-	-	-	-	-	-
**H18**	2.39E-01	2.23	2.94	0.10	2.58E-01	2.20	2.90	0.27
**T19**	1.91E-01	1.98	2.60	0.08	1.46E-01	2.09	2.75	0.18
**V20**		-	-			-	-	
**E21**	-	-	-	-	-	-	-	-
**K22**	-	-	-	-	-	-	-	-
**G23**	-	-	-	-	-	-	-	-
**G24**	-	-	-	-	-	-	-	-
**K25**	-	-	-	-	-	-	-	-
**H26**	-	-	-	-	-	-	-	-
**K27**	1.68E+00	1.05	1.38	0.09	-	-	-	-
**T28**	-	-	-	-	-	-	-	-
**G29**	4.62E-01	1.42	1.87	0.09	8.39E-01	1.16	1.53	0.40
**P30**	-	-	-	-	-	-	-	-
**N31**	-	-	-	-	-	-	-	-
**L32**	2.87E-03	3.18	4.20	0.84	7.65E-03	2.76	3.64	0.57
**H33**	1.05E-02	3.13	4.13	0.52	1.63E-02	2.94	3.88	0.35
**G34**	2.41E-01	2.08	2.74	0.17		-	-	
**L35**	2.04E-02	2.24	2.95	0.57	5.56E-02	1.81	2.38	0.14
**F36**	1.86E-02	2.26	2.98	0.31	3.23E-02	2.02	2.66	0.17
**G37**	4.41E-02	2.35	3.10	0.10	3.33E-01	1.47	1.94	0.38
**R38**	1.25E-02	2.85	3.76	0.44	1.76E+00	0.70	0.93	0.52
**K39**	-	0.43	-	0.13-	-	-	-	-
**T40**	9.00E-02	1.87	2.47	0.07	-	-	-	-
**G41S**	1.70E-01	1.85	2.44	0.12	-	-	-	-
**Q42**	1.57E-01	1.74	2.29	0.08	-	-	-	-
**A43**	1.72E+00	0.68	0.90	0.08	-	-	-	-
**P44**	-	-	-	-	-	-	-	-
**G45**	1.23E+00	0.73	0.96	0.13	7.44E-01	0.95	1.25	0.47
**Y46**	-	-	-	-	-	-	-	-
**S47**	-	-	-	-	-	-	-	-
**Y48**	-	-	-	-	-	-	-	-
**T49**	1.22E+00	0.70	0.92	0.09	-	-	-	-
**A50**	-	-	-	-	-	-	-	-
**A51**	1.97E-01	1.50	1.98	0.09	-	-	-	-
**N52**	7.55E-01	1.22	1.60	0.07	-	-	-	-

**K53**	9.01E-01	1.01	1.33	0.10	-	-	-	-
**N54**	1.80E+00	0.91	1.20	0.10	-	-	-	-
**K55**	-	-	-	-	-	-	-	-
**G56**	-	-	-	-	-	-	-	-
**I57**	1.24E-01	1.37	1.80	0.07	-	-	-	-
**I58**	3.28E+00	-0.29	-0.39	0.17	1.35E+00	0.09	0.12	0.69
**W59**	2.36E-03	3.04	4.01	0.84	9.48E-02	1.44	1.89	0.21
**G60**	4.66E-03	3.23	4.25	0.74	1.32E-02	2.78	3.66	0.40
**E61**	-	-	-	-	-	-	-	-
**D62**	-	-	-	-	-	-	-	-
**T63**	-	-	-	-	-	-	-	-
**L64**	1.58E-03	3.37	4.44	0.91	2.40E-03	3.19	4.20	0.87
**M65**	4.78E-03	2.99	3.94	0.74	6.30E-03	2.87	3.78	0.68
**E66**	2.13E-01	1.68	2.22	0.07	3.61E-01	1.45	1.91	0.19
**Y67**	4.67E-03	3.20	4.22	0.77	6.88E-03	3.03	4.00	0.67
**L68**	8.23E-04	3.56	4.70	0.95	-9.31E-04	-	-	1.09
**E69**	8.48E-04	3.89	5.12	0.91	1.79E-03	3.56	4.70	0.92
**N70**	1.61E-01	2.12	2.79	0.05	2.80E-01	1.88	2.48	0.14
**P71**	-	-	-	-	-	-	-	-
**K72**	-	-	-	-	-	-	-	-
**K73**	4.66E-01	1.18	1.55	0.33	2.05E+00	0.54	0.71	0.28
**Y74**	1.38E-02	2.57	3.39	0.44	1.29E-01	1.60	2.11	0.20
**I75**	4.34E-03	2.75	3.63	0.75	6.74E-02	1.56	2.06	0.18
**P76**	-	-	-	-	-	-	-	-
**G77**	-	-	-	-	-	-	-	-
**T78**	4.55E+00	0.20	0.27	0.28	-	-	-	-
**K79**	9.35E-01	0.92	1.22	0.14	-	-	-	-
**M80**	1.05E+00	0.85	1.11	0.13	-	-	-	-
**I81**	-	-	-	-	-	-	-	-
**F82**	-	-	-	-	-	-	-	-
**V83**	-	-	-	-	-	-	-	-
**G84**	-	-	-	-	-	-	-	-
**I85**	2.90E-01	1.00	1.32	0.09	2.56E-01	1.05	1.39	0.20
**K86**	-	-	-	-	-	-	-	-
**K87**	-	-	-	-	-	-	-	-
**K88**	-	-	-	-	-	-	-	-
**E89**	-	-	-	-	-	-	-	-
**E90**	5.60E+00	0.43	0.57	0.26	-	-	-	-
**R91**	7.08E-03	3.23	4.26	0.67	5.50E-03	3.34	4.40	0.72
**A92**	1.20E-02	2.85	3.76	0.67	1.21E-02	2.85	3.75	0.45
**D93**	2.08E-02	2.90	3.82	0.25	2.09E-02	2.89	3.81	0.26
**L94**	9.07E-04	3.85	5.08	0.99	-1.33E-03	-	-	1.12
**I95**	3.10E-03	2.74	3.61	0.79	1.78E-03	2.98	3.93	0.91
**A96**	1.52E-03	3.48	4.58	0.91	-4.18E-04	-	-	1.05
**Y97**	1.90E-03	3.36	4.42	0.91	-1.62E-04	-	-	1.02
**L98**	2.15E-03	3.15	4.15	0.86	-5.25E-04	-	-	1.03
**K99**	1.89E-04	4.37	5.76	0.99	-1.68E-03	-	-	1.13
**K100**	5.77E-02	2.09	2.75	0.05	5.20E-02	2.13	2.81	0.13
**A101**	7.25E-02	2.01	2.65	0.06	8.19E-02	1.96	2.58	0.16
**T102**	2.50E-01	1.36	1.79	0.05	2.98E-01	1.28	1.69	0.17
**N103**	3.79E+00	0.63	0.84	0.15	-	-	-	-
**E104**	-	-	-		-	-	-	-

Table S2: Free energies of denaturation in water (kcal mol^{-1}) [and m values, kcal mol^{-1} M^{-1}] for WT H-ferricyt and the G41S variant.

Protein	pH 7, 20 °C [6]	pH 6.5, 15 °C (this work)
WT	10.5 (0.2) [4.4 (0.4)]	10.1 (0.4) [3.9 (0.5)]
G41S	7.9 (0.3) [3.0 (0.3)]	6.8 (0.5) [3.0 (0.3)]

Table S3: Backbone dynamic parameters for H-ferricyt WT and G41S variant derived from Model-free analysis.

	WT										G41S									
**Res.**	**M.**	**S^2**	**S^2 error**	**S^2_f**	**S^2_f error**	**τ_e (ps)**	**τ_e error**	**R_{ex} (s^{-1})**	**R_{ex} error**	**S.S.E.**	**M.**	**S^2**	**S^2 error**	**S^2_f**	**S^2_f error**	**τ_e (ps)**	**τ_e error**	**R_{ex} (s^{-1})**	**R_{ex} error**	**S.S.E.**
G1																				
D2	5	0.756	0.011	0.844	0.009	1189.20	92.13			0.00	1	0.798	0.015							3.83
V3	1	0.899	0.003							21.32	1	0.85	0.011							183.80
E4	1	0.932	0.002							11.59	5	0.846	0.015	0.924	0.009	900.71	90.87			0.00
K5	1	0.92	0.003							91.99	1	0.867	0.008							36.09
G6	1	0.97	0.003							165.40	1	0.986	0.007							33.47
K7	1	0.965	0.004							121.20	1	0.948	0.01							11.04
K8										0.00	1	0.934	0.005							48.25
I9	3	0.928	0.006					0.93	0.12	1.05	2	0.96	0.01			40.82	42.23			1.75
F10	1	0.964	0.006							98.79	1	0.97	0.009							0.82
I11	1	0.936	0.005							104.80	1	0.949	0.016							1.58
M12	1	0.93	0.003							34.24	2	0.95	0.009			25.35	6.79			0.23
K13	1	0.897	0.005							85.64	2	0.883	0.014			7.80	1.42			1.84
C14	1	0.982	0.011							194.20	1	0.989	0.014							17.42
S15	1	0.863	0.003							33.53	2	0.866	0.008			11.87	0.94			0.13
Q16	1	0.948	0.006							52.27	1	0.964	0.019							11.98
C17	1	0.852	0.013							9.76	1	0.859	0.012							65.81
H18	3	0.87	0.008					1.27	0.32	2.64	3	0.883	0.015					2.11	0.49	0.05
T19	2	0.881	0.005			17.41	2.91			0.06	1	0.884	0.015							17.03
V20	3	0.925	0.009					13.51	1.00	1.68	3	0.959	0.023					13.71	0.50	2.24
E21																				
K22	2	0.911	0.009			13.05	2.69			3.68	1	0.904	0.015							21.97
G23	4	0.965	0.018			53.97	210.19	15.53	0.50	0.00	3	0.986	0.038					15.82	1.20	0.00
G24	3	0.963	0.008					1.80	0.17	2.22	1	1	0.016							1.11
K25	3	0.909	0.006					2.43	0.10	2.13	1	1	0.008							35.05
H26	1	0.912	0.004							6.44	3	0.957	0.023					1.94	0.81	0.74
K27	1	0.889	0.009							22.14	2	0.899	0.022			36.13	17.56			1.06
T28																				
G29	1	0.962	0.008							49.41	1	0.938	0.033							7.21
P30																				
N31	1	0.889	0.008							28.89	1	0.941	0.022							18.85
L32	1	0.901	0.009							2.17	2	0.929	0.012			16.01	3.63			0.01
H33											3	1	0.013					12.23	2.03	0.16
G34											1	1	0.035							28.39
L35	1	0.958	0.005							31.30	2	0.95	0.011			32.87	45.20			0.32
F36	5	0.835	0.02	0.878	0.012	1448.30	227.10			0.00	1	0.943	0.017							11.37
G37	1	0.921	0.004							178.00	3	0.901	0.028					4.92	0.53	0.74
R38	1	1	0.003							3.19	1	1	0.011							12.76
K39	4	0.911	0.004			44.14	2.80	0.80	0.10	0.00	4	0.956	0.016			94.55	167.37	3.64	0.57	0.00

T40	1	0.94	0.004							22.01										
**G41S**	3	0.925	0.012					1.46	0.22	3.63	3	0.92	0.034					22.60	1.10	0.56
Q42	1	0.875	0.004							135.00										
A43	1	0.963	0.007							0.75	2	0.947	0.022			27.47	150.90			0.00
P44																				
G45	3	0.922	0.003					1.66	0.11	1.76	3	0.875	0.036					1.63	0.59	0.02
Y46	1	0.917	0.003							15.62	2	0.886	0.021			10.79	2.87			0.75
S47	5	0.815	0.01	0.862	0.01	1268.60	248.72			0.00	1	1	0.013							118.90
Y48	1	0.871	0.005							35.55	2	0.915	0.015			14.46	3.49			0.03
T49	1	0.92	0.004							22.91	3	0.914	0.026					1.22	0.42	0.70
A50	1	0.93	0.015							22.33	1	0.989	0.016							0.71
A51	1	0.935	0.003							51.02	1	1	0.006							117.20
N52	1	0.925	0.004							25.51	1	1	0.011							78.70
K53	2	0.944	0.004			16.03	5.05			1.96	3	0.952	0.031					9.10	0.50	0.65
N54	1	0.902	0.005							173.00										
K55	1	0.924	0.004							150.10	3	0.951	0.036					10.77	0.76	0.05
G56	1	0.971	0.014							162.80										
I57	4	0.828	0.005			31.30	1.38	2.46	0.47	0.00										
I58	1	0.906	0.003							126.30	4	0.89	0.015			23.12	7.06	12.37	0.34	0.00
W59	4	0.937	0.008			15.52	2.41	1.15	0.20	0.00										0.00
G60	3	0.924	0.012					1.03	0.21	0.69	1	0.939	0.012							2.10
E61	5	0.923	0.007	0.961	0.008	666.72	164.63			0.00	2	0.936	0.019			23.57	144.63			0.19
D62	5	0.79	0.007	0.857	0.004	1651.70	114.65			0.00	1	0.885	0.013							35.76
T63	1	0.951	0.006							34.04	1	0.88	0.008							47.65
L64	2	0.976	0.003			19.16	9.00			1.35	1	0.996	0.008							16.93
M65	1	0.976	0.005							54.40	1	0.956	0.007							0.95
E66	1	0.938	0.003							13.36	1	0.952	0.007							6.46
Y67	1	0.945	0.007							6.17	1	0.889	0.013							58.73
L68	1	0.962	0.004							28.98	1	0.97	0.014							18.02
E69	1	1	0.002							13.07	1	0.983	0.014							25.24
N70										0.00	1	0.866	0.01							14.00
P71																				
K72	5	0.864	0.011	0.91	0.006	604.73	73.90			0.00	1	0.938	0.007							168.90
K73										0.00	1	0.973	0.01							68.70
Y74											2	0.967	0.009			640.38	248.45			3.43
I75	1	0.839	0.005							20.02	1	0.885	0.01							6.31
P76																				
G77	2	0.942	0.011			26.75	8.22			0.51	3	0.928	0.03					10.58	0.55	0.10
T78	2	0.95	0.005			6.80	2.35			0.54	2	0.968	0.018			16.45	573.32			0.02
K79	1	0.968	0.009							12.84	1	1	0.015							10.36
M80										0.00	1	1	0.022							43.79
I81	1	0.975	0.019							14.74	1	1	0.024							1.33
F82	2	0.869	0.013			64.81	9.83			0.13	2	0.905	0.028			40.05	47.83			0.21
V83	2	0.922	0.019			50.69	62.58			0.19	2	0.927	0.029			48.75	186.35			2.85

G84											1	0.812	0.027							14.76
I85	1	0.894	0.003							2.98	1	0.888	0.009							2.19
K86	1	0.938	0.016							111.60	1	0.921	0.023							44.69
K87	5	0.873	0.007	0.929	0.004	585.97	48.83			0.00	5	0.83	0.017	0.912	0.016	826.59	156.86			0.00
K88	1	1	0.006							1.64	1	0.971	0.02							0.71
E89	2	0.985	0.003			114.70	102.66			0.19	1	0.964	0.015							0.88
E90	1	0.922	0.004							97.22	1	0.869	0.011							23.07
R91	1	0.93	0.004							15.87	1	0.92	0.01							4.30
A92	1	0.952	0.004							4.50	1	0.939	0.013							3.28
D93	1	0.943	0.006							12.38	1	0.96	0.012							3.83
L94	1	0.947	0.003							10.14	1	0.934	0.01							0.46
I95	3	0.936	0.004					1.19	0.13	4.09	1	0.97	0.01							1.72
A96	1	0.949	0.002							90.83	1	0.937	0.014							48.13
Y97	3	0.943	0.005					0.66	0.10	0.21	1	0.971	0.008							15.31
L98	1	0.989	0.004							127.30	1	0.981	0.007							82.49
K99	5	0.901	0.01	0.93	0.006	1463.40	167.09			0.00	1	0.938	0.009							106.60
K100	1	0.921	0.002							58.80	1	0.916	0.007							7.50
A101	4	0.939	0.005			18.75	1.89	3.42	0.10	0.00	1	1	0.005							141.50
T102	1	0.889	0.007							2.78	1	0.878	0.016							18.93
N103										0.00										0.00
E104	2	0.894	0.009			180.05	58.89			0.00	2	0.814	0.018			60.71	32.56			0.15
